# The significance of Brf1 overexpression in human hepatocellular carcinoma

**DOI:** 10.18632/oncotarget.6668

**Published:** 2015-12-18

**Authors:** Qian Zhong, Shaoyan Xi, Jianzhong Liang, Ganggang Shi, Yi Huang, Yanmei Zhang, Daniel Levy, Shuping Zhong

**Affiliations:** ^1^ State Key Laboratory of Oncology in South China, Sun Yat-sen University Cancer Center, Guangzhou, China; ^2^ Shantou University Medical College, Shantou, Guangdong, China; ^3^ Department of Biochemistry and Molecular Biology, Keck School of Medicine, University of Southern California, Los Angeles, CA, USA

**Keywords:** Brf1, Pol III genes, hepatocellular carcinoma, survival, alcohol

## Abstract

Brf1 (TFIIB-related factor 1) plays a crucial role in cell transformation and tumorigenesis. However, the significance of Brf1 expression in human HCC (hepatocellular carcinoma) cases remains to be addressed. In this study, biopsies of human HCC, liver tumor samples of mice and cell lines of normal and tumor liver were utilized to determine the alteration of Brf1 expression using cytological and molecular biological approaches. Brf1 expression is increased in human HCC cases, which is correlated with shorter survival times. Levels of Brf1 and Pol III (RNA polymerase III-dependent) gene transcription in HCC patients with alcohol consumption are higher than the cases of non-HCC with or without alcohol intake. Induction of Brf1 and Pol III genes by ethanol in hepatoma cells is higher than in non-tumor cells. Ethanol increases the rate of cell transformation. Repression of Brf1 inhibits alcohol-promoted cell transformation. Alcohol consumption enhances Brf1 expression to promote cell transformation. These studies demonstrate that Brf1 is a new biomarker of HCC.

## INTRODUCTION

Alcohol-induced liver injury, including liver steatosis, inflammation, fibrosis and cirrhosis, increases the risk of development of HCC (hepatocellular carcinoma) [1]. Alcohol combines with viruses (hepatitis B or C), carcinogens (aflatoxin), obesity, or diabetes mellitus to promote liver cancer development [2–5]. However, the mechanisms of alcohol-induced HCC and the significance of Brf1 (TFIIB-related factor 1) expression in HCC remain to be elucidated. Previous studies have shown that chronic alcohol consumption results in the production of acetaldehyde. Acetaldehyde is a by-product of alcohol metabolism catalyzed by ADH (alcohol dehydrogenase), which has direct mutagenic and carcinogenic effects *in vitro* and *in vivo* [6–8]. Although the exact mechanism by which alcohol causes HCC is still unclear, alcohol consumption is thought to induce liver carcinogenesis through various mechanisms: mutagenesis by the ethanol metabolite acetaldehyde, and oxidative damage, as well as by affecting the one-carbon metabolic pathways through reduced folic acid intake and use. However, little is known about the role of alcohol in Brf1 and Pol III gene (RNA polymerase III-dependent gene) transcription, which is responsible for protein synthesis and tightly linked to cell transformation and tumor development.

RNA Pol III is responsible for the synthesis of a variety of small untranslated RNAs, including 5S rRNA and tRNAs, which are elevated in both transformed and tumor cells suggesting that it plays a crucial role in tumorigenesis [9–17]. Brf1 is a key transcription factor, which specifically regulates Pol III gene transcription. Our studies have demonstrated that alcohol induces Brf1 expression and Pol III gene transcription in both *in vivo* and *in vitro* [11]. Liver tumor development was induced in alcohol-fed NS5A (HCV non-structural 5A protein) transgenic mice [12]. Ethanol increased the expression of TFIIIB subunits, TBP, Brf1 and Bdp1 in HepG2-ADH cells [11]. Regulation of Bdp1, but not Brf1, occurred through alterations in TBP expression [13]. A repression in Brf1 expression decreased Pol III gene transcription and was sufficient to inhibit cell transformation [14–17]. Brf1 induction caused an increase in cell proliferation and oncogenic transformation [14–17]. However, it is not clear what role of Brf1 overexpression plays in human cancers. Therefore, in the present studies, we have determined the alteration of Brf1 expression and analyzed the significance of its change in human HCC cases. The results indicate that Brf1 is overexpressed in HCC cases, which results in a shorter overall survival period. Brf1 expression and Pol III gene transcription in HCC cases with alcohol consumption are further increased. Ethanol elevates rate of cell transformation, whereas repression of Brf1 reduces the ethanol-increased the rate of cell transformation. These studies demonstrate that Brf1 is a novel biomarker of HCC, which play critically important role in HCC development.

## RESULTS

### The expression of Brf1 in the biopsies of HCC patients

**Figure 1:**
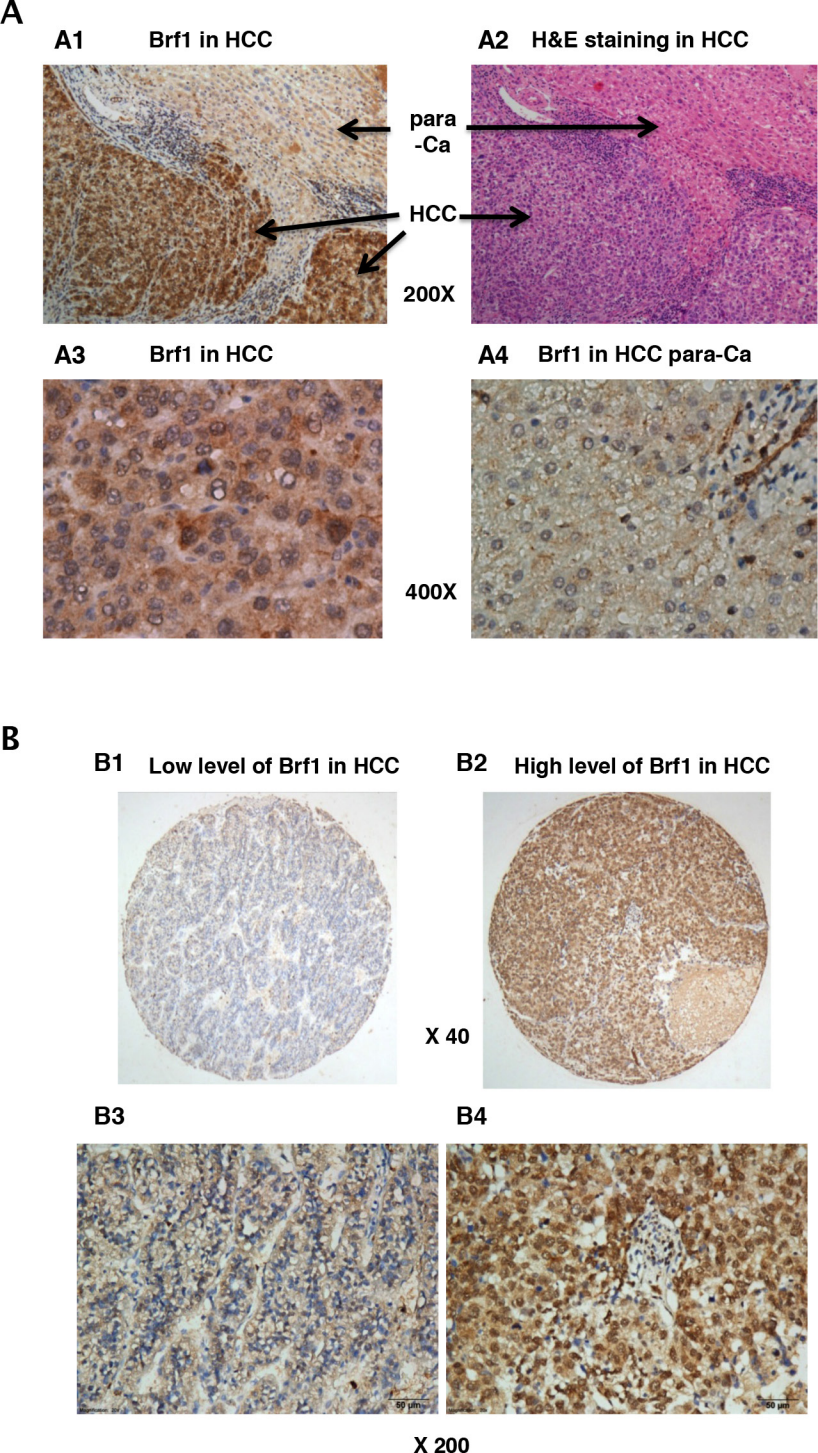
Immunohistochemistry (IHC) staining of Brf1 in human HCC **A.** *Brf1 staining.* (A1) IHC staining of Brf1 in HCC; (A2) H&E staining of HCC liver tissue; (A3) IHC staining of Brf1 in cancer foci of HCC; (A4) IHC staining of Brf1 in the para-Ca tissue of HCC. (A1 & A2) 200 X magnification; (A3 & A4) 400 X magnification. A representative staining of Brf1 in HCC. **B.** *Weak and strong staining of Brf1* (B1 & B3) are low expression of Brf1 in HCC; (B2 & B4) are high expression of Brf1. (B1 & B2) 40 x magnification; (B3 & B4) 200 x magnification.

**Figure 2:**
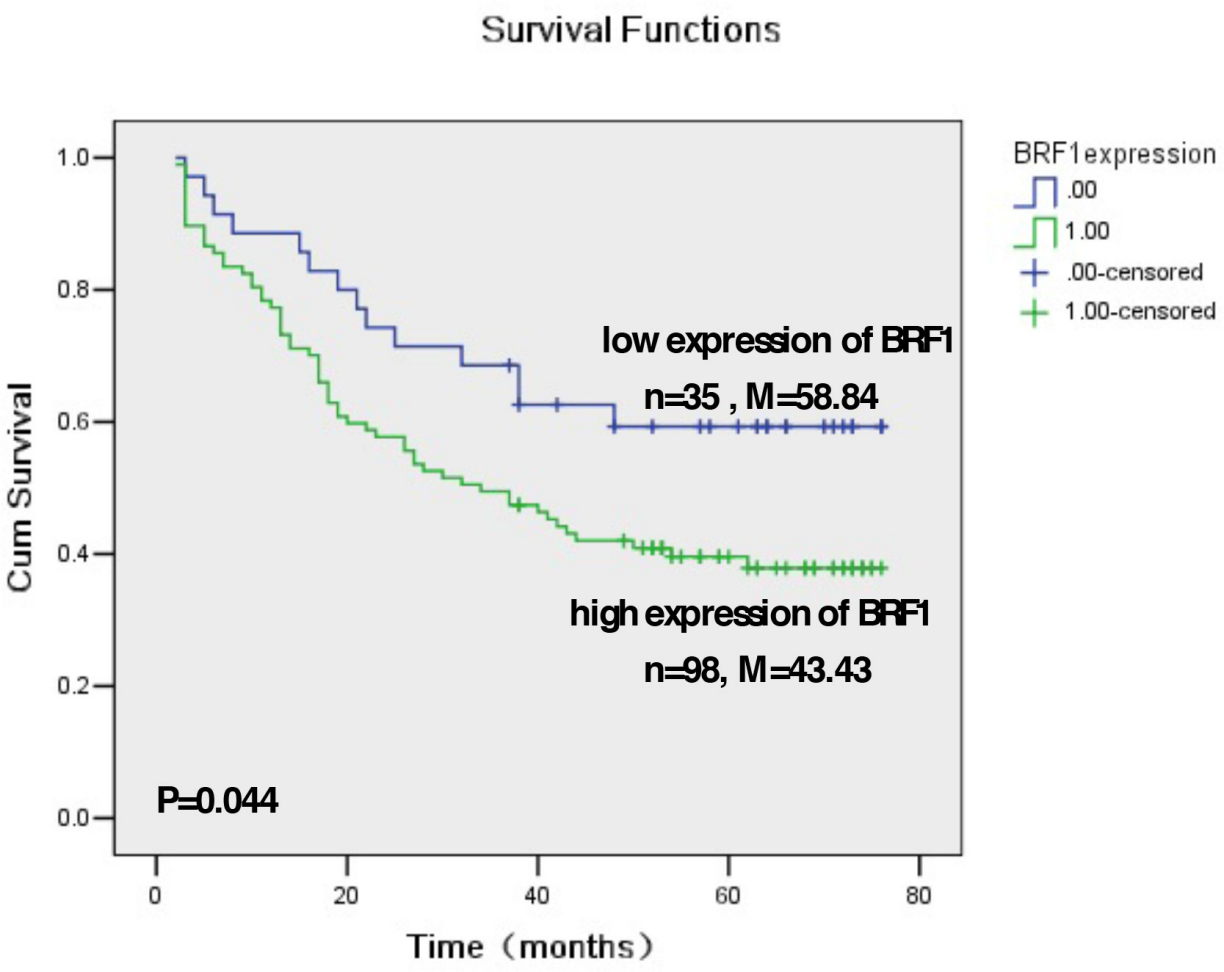
Kaplan-Meier survival curve and log-rank test analysis of the association between Brf1 expression and HCC patient survival **A.** Brf1 expression of 133 HCC cases wasdetermined by pathological analysis and immunohistochemistry staining. n = number of patients in the subgroup, M = median survival in months of the subgroup.

Our studies have demonstrated that alcohol induced Brf1 expression and Pol III gene transcription *in vitro* and *in vivo* by using cell culture models and NS5A transgenic mice [11, 14–17]. To investigate the significance of Brf1 expression in HCC patients, we have carried out immunohistochemistry (IHC) analysis using a specific antibody against Brf1 in tissue microarrays of 133 human HCC tissue samples. The clinical pathological characteristics of HCC cases were summarized in (Supplementary Table S1). The representative pictures of IHC are shown in Figure 1. Strong signals of Brf1 staining are observed in HCC tissue, compared to para HCC tissue (Figure 1A and 1B). The histological study indicates the appearance of Brf1 in the tissues of HCC and para HCC (Figure 1A1, 3). The result reveals that the localization of Brf1 is primarily in the nucleus and cytoplasm in HCC cells with only weak signal of Brf1 detected in para HCC tissue (Figure 1A2, 4). We observed staining of BRF1 in all of 133 HCC cases. All the samples were separated into either low or high BRF1 expression groups (Figure 1B). High expression of BRF1 was observed in 98/133 (73.5%) of HCC samples. The IRS (immunoreactivity score) was multiplied by the score of tumor cell percentage and the score of staining intensity. Brf1 expression was significantly associated with serum AFP (alpha-fetoprotein) values (P=0.028, Supplementary Table S2) in all HCC patients. However, there was no significant correlation between Brf1 expression and other clinic pathological features, such as patient age, gender, TNM stages, recurrence (P>0.05, Supplementary Table S2). Univariate Cox proportional hazard regression analysis showed that Brf1 expression (P<0.001), tumor diameter (P=0.007), recurrence (P=0.003), serum AFP (P=0.011) and TNM stages (P<0.001) were significantly associated with overall survival (Table 1). To determine whether BRF1 expression was correlated with OS (overall survival) of HCC patients, we evaluated the prognostic value of Brf1 through estimation of OS using Kaplan-Meier and log-rank test analyses. As shown in Figure 2, high Brf1 expression was significantly related to poor OS compared to low Brf1 expression (43.43 versus 53.84, P=0.044). We analyzed the RNA-seq and tumor progression data from “Liver Hepatocellular Carcinoma (TCGA, provisional)” at cBioPortal [18–19] and found that high expression of BRF1 predicts a poor overall survival (20.6 versus 53.3 months, P=0.032, Supplementary Figure S1).

**Table 1:** Association of various factors with overall survival in 133 HCCs determined by COX regression model

Univariate analysis	Multivariate analysis			
Variable	HR_a_(95%CI_b_)	*P_c_*	HR(95%CI)	*P*
Gender Male vs. Female	0.728(0.406-1.305)	0.286		
Age ≤50yr vs. >50yr	1.278(0.803-2.033)	0.301		
Tumor diameter ≤5cm vs. >5cm	2.060(1.217-3.486)	0.007	1.364(0.779-2.386)	0.277
Serum AFP (ng/ml) <400 vs>400	1.810(1.142-2.867)	0.011		
Serum ALT <40 vs>40	1.123(0.767-1.921)	0.409		
Recurrence Absent vs. Present	2.281(1.730-4.598)	0.003	1.742(1.010-3.005)	0.046
TNM stage Early vs. Advanced	4.032(2.512-6.472)	0.000	2.760(1.595-4.775)	0.000
BRF1 expression Low vs. High	1.823(1.018-3.267)	0.044	1.470(0.812-2.663)	0.204

### Alcohol-increased Brf1 expression promotes liver tumor development

**Figure 3:**
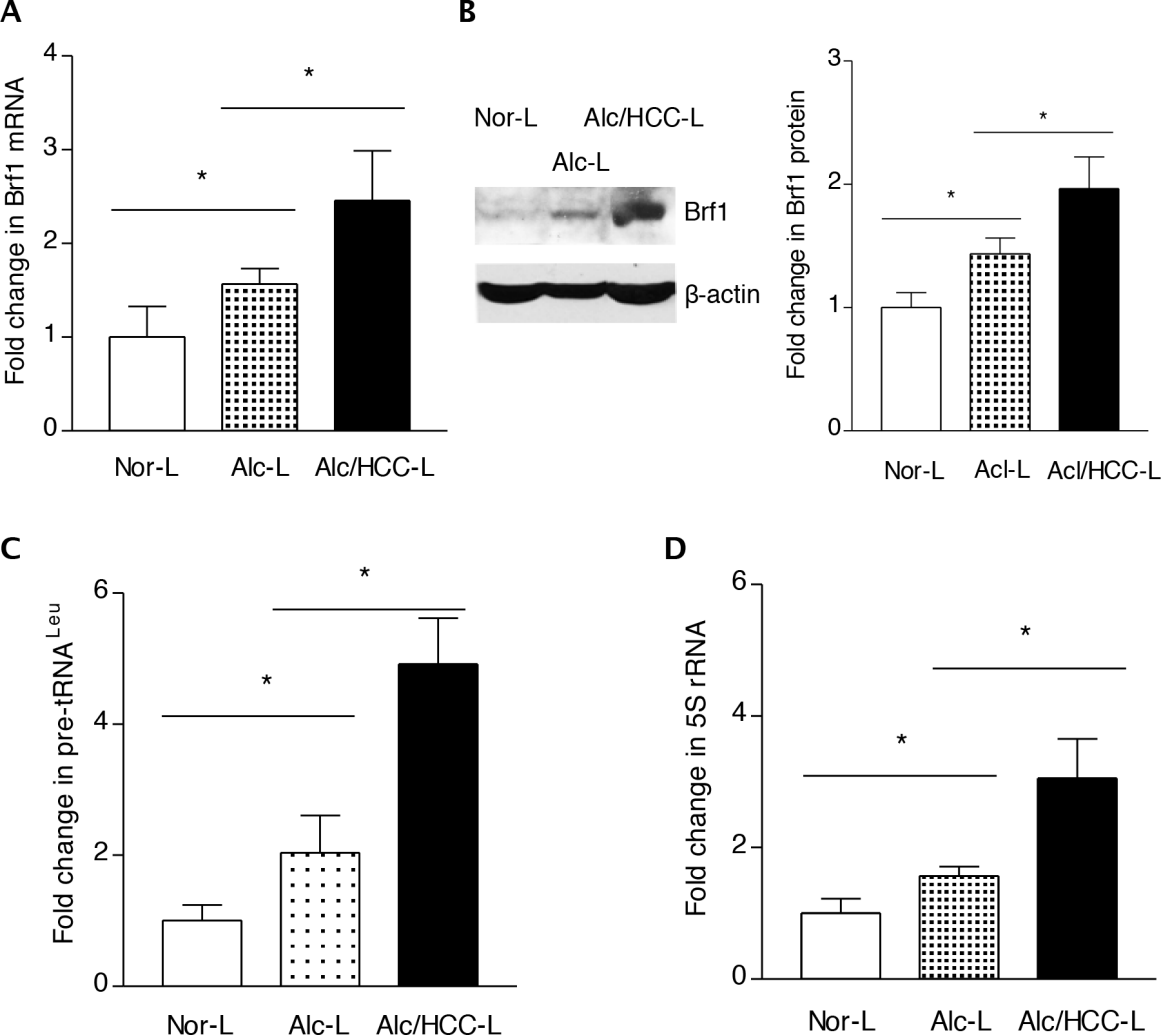
Expression of Brf1 and Pol III genes in HCC cases with alcohol consumption The total RNA and tissue lysates were extracted from the human liver tissues of normal (Nor-L, n=6), alcohol consumption (Alc-L, n=8) and HCC cases with alcohol consumption (Alc/HCC-L, n=7). Immunoblot analysis was performed to determine cellular levels of Brf1 protein (right) and its quantify data (left) **B.** RT-qPCR was carried out to measure the amounts of Brf1 mRNA **A.** pre-tRNA^Leu^ **C.** 5S rRNA **D.** The transcription levels of the genes were calculated by normalizing to the amount of GAPDH mRNA. The fold change was normalized by Nor-L. The bars represent Mean ± SE of at least three independent determinations. *: p<0.05 as indicated.

The above studies have demonstrated that high expression of Brf1 in HCC tissues is correlated to the short overall survival times of the human HCC cases. To investigate the role of alcohol-induced deregulation of Brf1 and Pol III genes in HCC, we further explore the relationship of alcohol consumption with Brf1 expression. The cellular levels of Brf1 protein and mRNA were determined in human normal livers (Nor L), alcohol consumption liver (Acl-L) and alcohol consumption-HCC liver (Alc/HCC-L). The results indicate that protein and mRNA levels of Brf1 are increased in Alc/HCC-L, compared to the levels observed in Nor-L and Acl-L (Figure 3A-3B). In addition, the amounts of precursor tRNA^Leu^ and 5S rRNA transcript of these samples were measured by RT-qPCR. The results reveal that the levels of Pol III genes, both pre-tRNA^Leu^ (Figure 3C) and 5S rRNA (Figure 3D) are increased in Acl/HCC-L, compared to the transcription levels of these genes in the samples of Nor-L and Acl-L.

**Figure 4:**
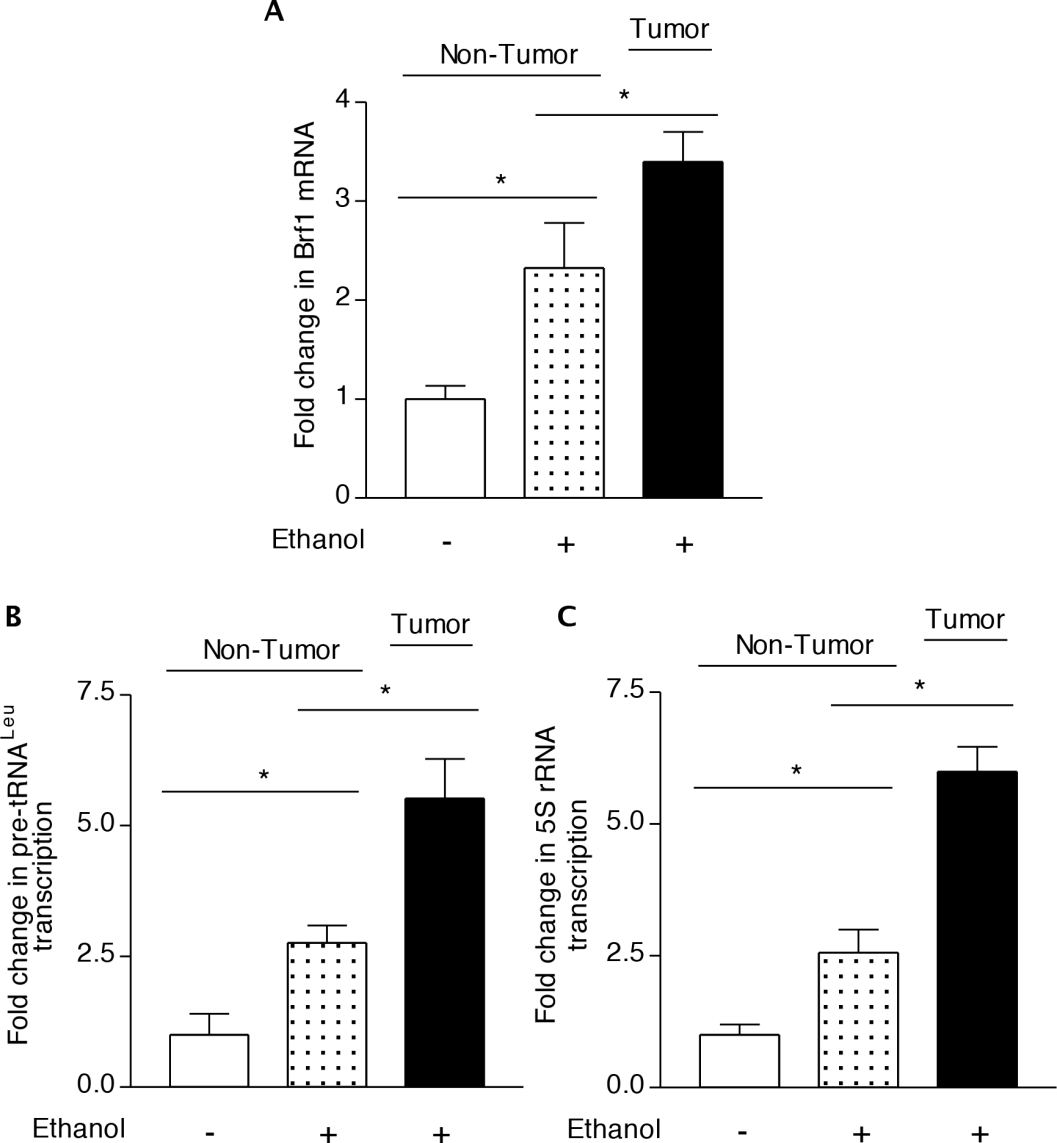
Transcription of Brf1 and Pol III genes in chronic alcohol administration in mice C57BL/6 transgenic mice harboring the HCV NS5A gene were fed with 3.5% ethanol in the Lieber-DeCarli liquid diet or control diet for 12 months. Liver tissues were harvested from these mice. RNA was extracted from the non-tumor or tumor portion of the livers and RT-qPCR was used to measure the amounts of Brf1 **A.** pre-tRNA^Leu^ **B.** 5S rRNA **C.** relative to GAPDH. The values represent means ± SE from three independent experiments. Each group of mice includes at least five mice. The fold change was calculated in each group by normalizing to the mice fed with control diet. *: p<0.05 as indicated.

To further explore the mechanism of alcohol-promoted liver tumor formation, we utilized the liver tissues (kindly provided by Dr. Machida K) of hepatitis C virus (HCV) non-structure 5A (NS5A) transgenic mouse to determine the role of Brf1 in this process. The results indicate that the precursor tRNA^Leu^ and 5S rRNA transcript levels are elevated in HCC tissues of alcohol-fed NS5A mice, compared to the levels of the Pol III genes in the liver tissues from non-alcohol-fed and alcohol-fed NS5A mice (Figure 4B-4C). More interestingly, the cellular level of Brf1 mRNA in HCC tissues of alcohol-fed NS5A mice is also significantly higher than in the non-tumor liver tissues of these mice with or without alcohol intake (Figure 4A). These studies using human biopsy samples and mouse liver tissues demonstrate that increases in Brf1 and Pol III gene expression are tightly linked to alcohol-associated HCC.

### Difference of Brf1 expression in tumor and non-tumor liver cell lines and the role of Brf1 in alcohol-promoted cell transformation

**Figure 5:**
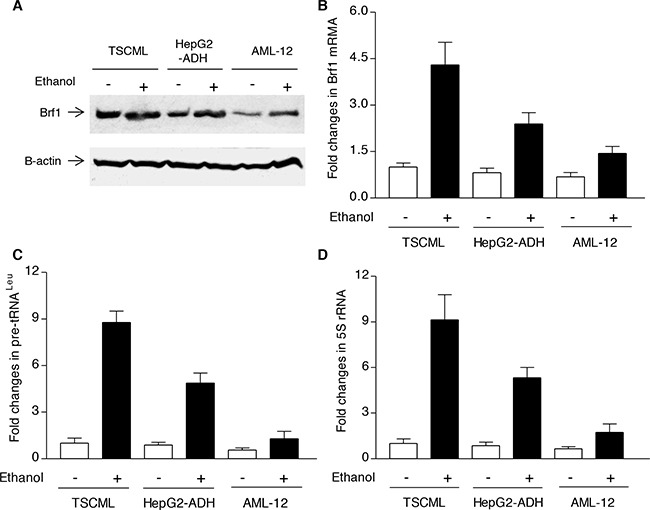
Brf1 and Pol III gene expression in non-tumor and tumor liver cell lines The cells of TSCML, HepG2-ADH and AML-12 cell lines were starved in DMEM-F12 for 4h. Cells were treated with or without 50 mM ethanol for another 1 h: The cell lysates were extracted from these cells to determine the levels of Brf1 protein by immunoblot analysis **A.** Total RNAs were extracted from these cells and RT-qPCR was performed to measure the amounts of Brf1 mRNA **B.** pre-tRNA^Leu^ **C.** and 5S rRNA **D.** The fold change was calculated by normalizing to the amount of GAPDH mRNA. The bars represent Mean ± SE of at least three independent determinations.

**Figure 6:**
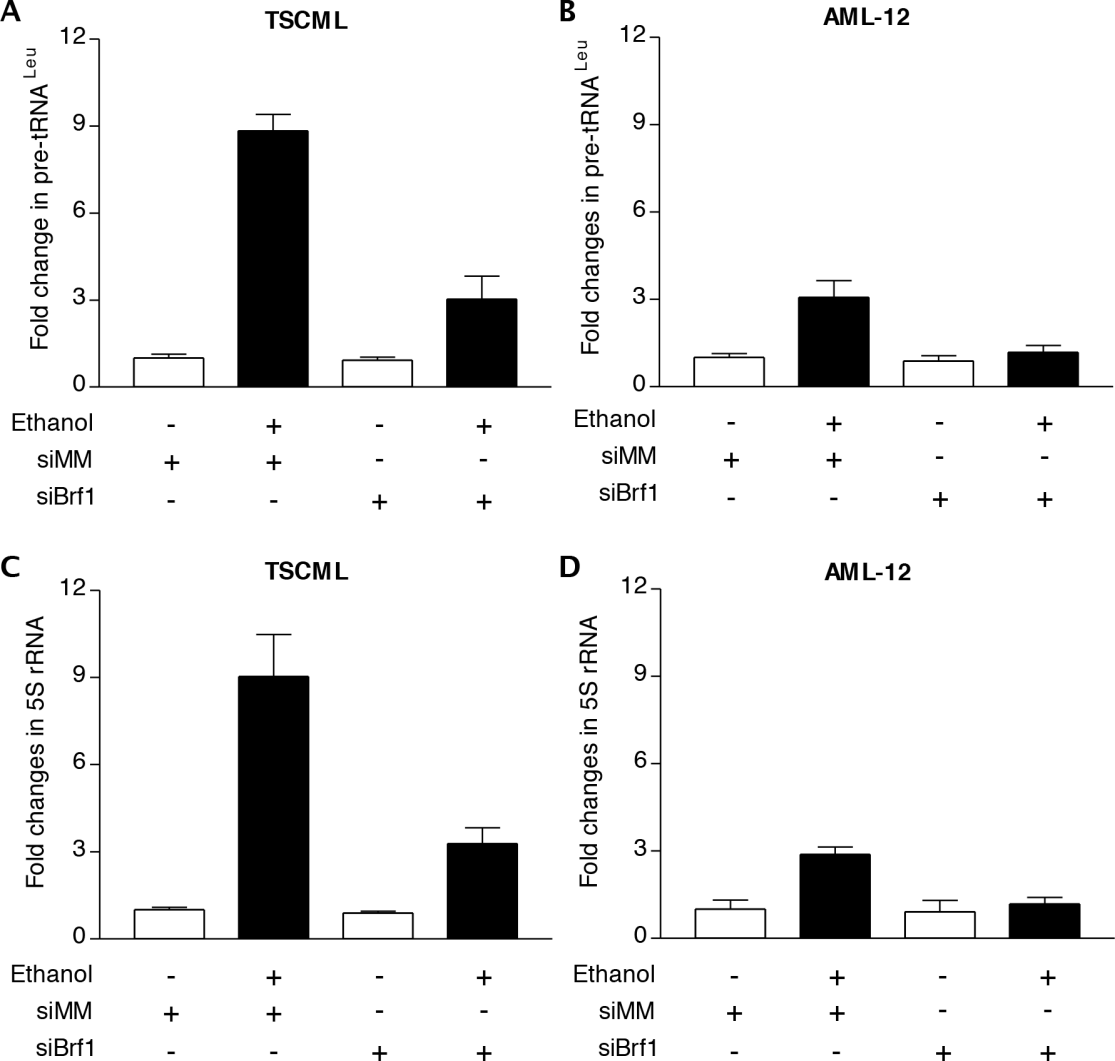
Repression of Brf1 decreases the induction of Pol III genes caused by alcohol The cells of TSCML and AML-12 were transfected with mismatch RNA (mmRNA) or Brf1 siRNA for 48 h. The cells were treated as described in Figure 5. Total RNAs were extracted from these cells and RT-qPCR was performed to measure the amounts of pre-tRNA^Leu^ **A, B.** and 5S rRNA **C, D.** The fold change was calculated by normalizing to the amount of GAPDH mRNA. The bars represent Mean ± SE of at least three independent determinations.

**Figure 7:**
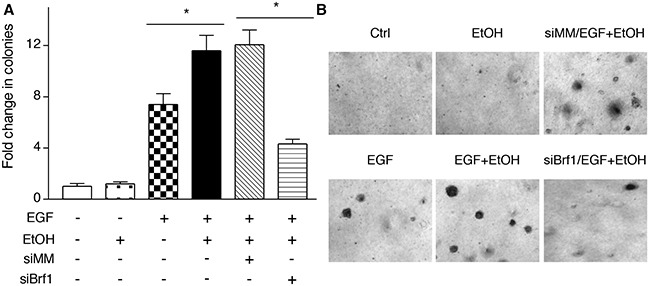
Reducing the amounts of cellular Brf1 inhibits alcohol-increased the rate of cell transformation AML-12 cells were poured in triplicate into 6-well plate with 0.35% agar containing 50 mM ethanol or PBS as control and grown in the medium with or without 50 mM ethanol and plus EGF (20ng/ml) for 4 weeks or longer. The cells were analyzed for colony formation in soft agar. Colonies were counted at 3-4 weeks after plating. Values are the means ± SE (n≥C3). *: p < 0.05 as indicated.

The studies from our laboratory and others have demonstrated that reduction of Brf1 expression decreases Pol III gene transcription [11, 14–17]. The above studies have demonstrated that Brf1 expression is increased in human HCC tissues, compared to the para tissues of HCC. Therefore, we further determined the cellular levels of Brf1 in tumor and non-tumor liver cell lines in these systems. The results reveal that the levels of Brf1 mRNA and protein in tumor cell lines [TSCML (tumor stem cells of mouse liver) and HepG2-ADH], are higher than the non-tumor line (AML-12), an immortalized liver cell line of mouse (Figure 5A and 5B). Furthermore, the induction of Brf1 expression caused by ethanol in tumor lines is significantly higher than in non-tumor line (Figure 5A and 5B). This suggests that oncogenic cell lines are more sensitive to the alcohol-induced response. In addition, we also found similar results for Pol III gene transcription in these cell lines (Figure 5C and 5D). These results demonstrate that alcohol-induced Brf1 and Pol III gene transcription in tumor lines is significantly higher than in non-tumor line. This suggests that higher levels of Brf1 are associated with the oncogenic status of these cells. Thus, we further determined whether repressing Brf1 affects the alcohol-induced phenotype. Our earlier studies have demonstrated that repression of Brf1 expression and Pol III gene transcription by Brf1 siRNA inhibits cell transformation [15–17, 20]. Here, we established whether alcohol affects the rate of cell transformation and repression of Brf1 inhibits alcohol-promoted transformation of AML-12 cells. Our studies have demonstrated that Brf1 siRNA efficiently decreases its cellular levels of protein and mRNA [15–17, 20]. Here, our results show that reduction of Brf1 by its siRNA inhibits precursor tRNA^Leu^ and 5S rRNA transcription in TSCML and AML-12 (Figure 6A-6D). To further assess the effect of Brf1 on alcohol-caused phenotypic alteration, we performed a soft agar assay. The results show that alcohol increases the rate of EGF-caused colony formation of AML-12 cells (Figure 7A), whereas Brf1 siRNA represses cell anchorage-independent growth (Figure 7A-7B). This indicates that the change of Brf1 levels in the cells results in alteration of alcohol-promoted phenotypes of non-tumor liver cells.

## DISCUSSION

Our results have indicated that Brf1 expression is increased in both human female and male cases of HCC. Further analysis reveals that human HCC cases with high expression of Brf1 have a shorter overall survival period. The cellular level of Brf1 in human HCC cases with alcohol consumption is higher than non-HCC cases with or without alcohol intake. Alcohol feeding promotes liver tumor development of NS5A mice, while Brf1 expression and Pol III gene transcription are increased in alcohol-fed NS5A mice. Furthermore, our studies demonstrate that the amounts of Brf1 and Pol III gene expression in tumor liver cell lines is significantly higher than non-tumor liver cell line. Repression of Brf1 expression reduces alcohol-increased rate of cell transformation. Together, these studies demonstrate that Brf1 is a novel biomarker of HCC, which plays a key role in alcohol-associated liver tumor development.

Studies have indicated that females are more resistant to several diseases, such as HCC [21–22]. HCC is the fifth common cancer and ranks third in annual mortality worldwide [23]. Women have a significantly lower incidence of HCC than men [23]. Diethylnitrosamine (DEN) administration caused HCC in 100% of male mice [24], but only in 10% of female mice [25]. Here, our results reveal this feature: 84% cases (111/133) of HCC are the males, but only 16% cases (21/133) are the females, which further supports the idea that females are resistant to HCC development. Because high expression of Brf1 is associated with shorter overall survival period (Figure 2 and Supplementary Figure S1), this implies that the human HCC cases with high expression of Brf1 have a poorer prognosis. Studies have indicated that Foxa1/2 deficient female mice are more susceptible to DEN-induced liver cancer development because of the loss of the interaction between Foxa1/2 and ERα [26]. A reason for the susceptibility for HCC development in female and male mice may depend on the interaction of sex hormones with factors, such as Foxa1/2, ERα or AR (androgen). The studies from our laboratory have demonstrated that DEN increases Brf1 expression and Pol III transcription in liver cells [16], whereas ERα upregulates alcohol-induced Brf1 expression in breast cells [15]. This suggests that a potential interaction of Foxa1/2, ERα and Brf1 may exist, which may modulate Pol III gene transcription. Given that high expression of Brf1 is associated with short overall survival times (Figure 2 and Supplementary Figure S1), reducing the cellular level of Brf1 of HCC patients may be used to extend overall survival period and measuring the level of Brf1 expression may be used to monitor the efficacy of treatment of HCC.

Oncogenic proteins, such as Ras, c-Jun, and c-Myc, stimulate Pol III gene transcription [10–11, 27–28], whereas tumor suppressors, such as pRb, p53, BRCA1 and PTEN repress transcription of this class of genes [10–11, 27–31]. Studies have indicated that RNA Pol III transcription products are elevated in both transformed and tumor cells suggesting that they play a crucial role in tumorigenesis [11, 14–17, 20]. Consistent with this idea, enhanced Pol III gene transcription is required for oncogenic transformation [13–17, 20]. The ability of these oncogenic and tumor suppressor proteins to regulate Pol III gene transcription result from their capacity to modulate the TFIIIB complex. The TFIIIB complex consists of TATA box-binding protein (TBP) and its associated factors, Brf1 and Bdp1. We have demonstrated that enhancement of Brf1 expression and Pol III gene transcription is correlated with liver tumor formation [11]. In contrast, repression of Brf1 expression inhibits cell transformation [14–16, 20]. Our studies indicate that alcohol increases expression of Brf1 and Pol III genes [11, 14, 20]. We have demonstrated that alcohol increases cellular level of c-Jun, which occupies to Brf1 promoter to enhance Brf1 expression and Pol III gene transcription [11]. Here, the results further indicate that the cases of human HCC with alcohol consumption reveal higher expression of Brf1 and Pol III genes, compared to other groups (Figure 3). The animal studies further suggest that alcohol feeding of mice promotes liver tumor formation (Figure 4). This shows that alcohol-increased expression of these genes is critically important during HCC development. Alcohol has been classified as carcinogenic in human [32–33]. Target sites for alcohol-related carcinogenesis in human include the breast, liver and multiple additional organs. Is there a common mechanism, which enhance*s* alcohol-associated cancer development in different human organs? Cancer cells have a consistent cytological feature of nucleolar hypertrophy, where RNA Pol III genes are transcribed [10]. This feature provides the possibility of elucidating a common mechanism of alcohol-associated human cancers by determining deregulation of Brf1 and Pol III genes. Animal experiments have shown that alcohol intake promotes tumor development [34, 35]. These studies are consistent with our findings in Figure 4. Furthermore, we have found that alcohol increases expression of Brf1 and Pol III genes in liver and breast cell lines and the cellular levels of Brf1 in cancer cell lines are higher than in non-tumor cell lines [11, 15–18, 28]. In contrast, repression of Brf1 expression inhibits cell transformation [11, 15–17, 20, 28]. The present studies indicate higher level of Brf1 in human tumor tissue of HCC. This suggests that the high level of Brf1 reflects the oncogenesis status of cells and Brf1 may be a novel biomarker for cancers, whereas alcohol-caused deregulation of Brf1 and Pol III genes may be a common mechanism of alcohol-associated cancers.

In summary, the present studies provide evidence that Brf1 is overexpressed in human HCC cases, where high expression of Brf1 results in shorter overall survival times. Alcohol intake increases cellular levels of Brf1 and Pol III genes expression to promote liver tumor development. Liver tumor cell lines show high levels of Brf1 protein and mRNA, compared to non-tumor cell lines. In contrast, repression of Brf1 decreases the induction of Pol III gene transcription caused by alcohol and reduces the rate of alcohol-induced cell transformation. These studies indicate that Brf1 could be a biomarker of diagnosis and prognosis of HCC. These findings suggest the possibility that detecting cellular level of Brf1 expression may be a potential approach to measure the efficacy of HCC treatment.

## MATERIALS AND METHODS

### Hepatocellular carcinoma patients and clinical tissue specimens

All 133 paraffin-embedded HCC specimens collected for immunohistochemistry (IHC) assay were clinically and histologically diagnosed at Sun Yat-sen University Cancer Center (SYSUCC), Guangzhou, China between 2005 and 2007. Tissue microarrays were constructed from each resected specimen. If the HCC is multiple, then the biggest tumor was selected in this tissue microarray. The clinicopathological characteristics are summarized in Supplementary Table S1. All the samples were obtained during the surgery prior to radiotherapy or chemotherapy. Cancer TNM stage was defined on the basis of the AJCC (American Joint Committee on Cancer). All the patients were followed from the date of diagnosis until death or the latest census date. The informed consent was obtained from each patient and the study was approved from the Institute Research Ethics Committee of Sun Yat-Sen University.

### Reagents and antibodies

Cell culture medium (DMEM/F12), OPTI-MEM, Lipofectamine 2000 and TRIzol reagent were from Life Technologies (San Diego, CA, USA). Antibody against β-actin was obtained from Santa Cruz Biotech (Santa Cruz, CA, USA). Mismatch RNA (mmRNA) was described previously [16]. Brf1 antibody was from Bethyl laboratories Inc (Montgomery, TX, USA). The sequences of primers and Brf1 siRNA were described in (Supplementary Table S3) [20]. Tumor stem cells of mouse liver (TSCML) were kindly provided by Dr. K. Machida at USC (University of Southern California). AML-12 cell line was from ATCC. HepG2-ADH cell line was kindly provided by Dr. D.L. Clemens (University of Nebraska). The animal experiments were conducted in accordance with the approved institutional animal care and use committee protocol at USC.

### Immunohistochemistry staining

Formalin-fixed, paraffin-embedded HCC tissue microarrays were cut into 4-μm thick sequential sections and baked at 65°C for 30 minutes. Deparaffinization of sections was performed with xylene two times and at different concentrations of alcohol and rehydrated. These sections were immersed in 3% hydrogen peroxide for 10 min to block endogenous peroxidase activity at room temperature, and then boiled in Citrate Antigen Retrieval Solution (PH=6.0) for 5 min in a microwaver for antigen retrieval. After that, the sections were incubated with diluted rabbit polyclonal anti-Brf1 antibody (1:200 dilutions, Bethyl, USA) overnight at 4°C and a secondary antibody for 30 min at 37°C. After the sections were incubated with DAB (3,3-diaminobenzidine) staining for 2 min for protein detection. The sections were counterstained with Mayer's hematoxylin to stain nucleus and were finally dehydrated and mounted. A negative control was obtained by replacing the primary antibody with a normal rabbit IgG.

### Evaluation of IHC

The immunoreactivities were scored separately by two pathologists blinded to the clinical parameters using H-score method. Tumor cell percentage were scored as 0~100% positive tumor cells. Staining intensity was categorized: 0, no staining; 1, weak staining; 2, moderate staining and 3, strong staining. The two individual parameters were multiplied. Then we can get an immunoreactivity score (IRS) ranging from 0 to 300%. All results were confirmed by at least 2 pathologists in a double-blind analysis. An optimal cut-off value for high and low expression was determined by the median of IRS. All HCC patients were divided into two groups according to the median score of anti-BRF1 immunostaining

### Real time quantitative PCR (RT-qPCR) and transfection

Liver cancer cell lines and non-tumor liver cell lines were grown to 85% confluence and starved in serum-free for 3-4 h. The cells were treated with 50 mM ethanol for another 1 h. Total RNAs of the cells and the liver tissues from human and mice were extracted with TRIzol reagent (Invitrogen). For siRNA transfection assays, AML-12 cells were cultured in 10% FBS/DMEM-F12 medium as described previously [17]. Serum-free medium was added to each dish with Lipofectamine-2000 Brf1 siRNA or mmRNA complexes, and cells were further incubated for 4 h at 37°C. The cells were incubated for 48 h before harvesting. Total RNA samples were quantified and reverse-transcribed. After first-strand cDNA synthesis, the real time qPCR (RT-qPCR) were performed with specific primers (Supplementary Table S4) and PCR reagent kits (Bio-Rad Biotech) in the ABI prism 7700 Sequence Detection System. Precursor of tRNA^Leu^ and 5S rRNA transcripts and Brf1 mRNA were measured by RT-qPCR as described previously [17].

### Cell anchorage-independent growth

*AML-12* cells (1 × 10^4^ cells/well in 6-well plate) were transfected with mismatch RNA (mmRNA) or Brf1 siRNA (Supplementary Table S3). The cells were suspended in 0.35% (w/v) agar in 10% FBS/DMEM/F12 with or without 50 mM ethanol, 20ng/ml EGF or both EGF and ethanol over a bottom layer of media with 0.5% (w/v) agar. Cells were fed fresh complete media with EGF or/and ethanol twice weekly. Colonies were counted 3-4 weeks or longer after plating as previously described [16].

### Immunoblot analysis

Cells were treated with 50 mM ethanol to extract total cell lysates. Protein concentrations of the resultant lysates were measured by the Bradford method using Fluostar Omega spectrometer (Cell Biology Core Laboratory of University of Southern California Research Center for Liver Diseases, P30 DK048522). Lysates (50 μg of protein) were subjected to immunoblot analysis as previously described [36–37]. Membranes were probed with specific antibodies as indicated. Hybond-P membrane was used for protein transfer. Bound primary antibody was visualized using horseradish peroxidase-conjugated secondary antibody (Vector Laboratories) and enhanced chemiluminescence reagents (Cell Signaling technology).

### Statistical analysis

Statistical analysis was performed using SPSS software, version 17.0 (SPSS, Chicago, USA). The paired T test was used to analyze the significant difference of Brf1 mRNA expression between HCC tissues and the paired adjacent normal tissues. Chi-square test was used to assess the association between Brf1 expression and clinicopathological status of HCC patients. Survival curves for both Brf1 high-expression and Brf1 low-expression patients were performed using the Kaplan-Meier analysis and the log-rank test for comparison. Univariate and multivariate regression analysis were performed with the Cox proportional hazards regression model to determine the effect of particular prognostic factors including Brf1 expression on death events. A value of P < 0.05 was considered statistically significant.

## SUPPLEMENTARY TABLES AND FIGURES



## Abbreviations

Brf1: TFIIB-related factor 1
HCC: hepatocellular carcinoma
Pol III genes: RNA polymerase III-dependent genes
IHC: immunohistochemistry
OS: overall survival
