# Supplementary material for: The significance of Brf1 overexpression in human hepatocellular carcinoma
Source: Oncotarget. 2015 Dec 18;7(5):6243–54. doi: 10.18632/oncotarget.6668 (PMC4868753; doi:10.18632/oncotarget.6668)
Supplement: Supplementary file 1 [file oncotarget-07-6243-s001.pdf]

SUPPLEMENTARY TABLES AND FIGURES

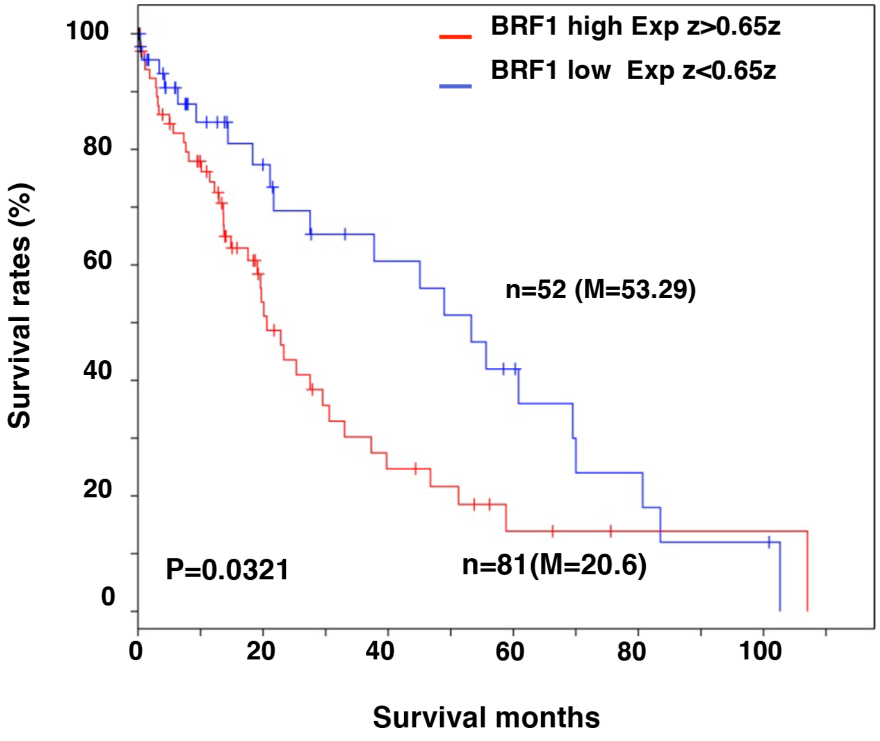

Supplementary Figure S1: Kaplan–Meier plot of Overall Survival for 133 Liver Hepatocellular Carcinoma from publicly available TCGA data at cBioPortal stratified by level of BRF1 expression. The Brf1 expression level z-score cut-off used for dividing the cases with high expression of Brf1 from the cases with low expression of Brf1. n = number of patients in the subgroup, M = median survival in months of the subgroup.

Supplementary Table S1: Characteristics of 133 HCC patients

Characteristics	HCC patients(N = 133)
Sex	
Female	21
Male	112
Age(years)	
≦50	62
> 50	71
Follow-up time(month)	
Median(range)	12(75)
Tumor size stage	
T1+T2	86
T3a+T3b	46
Clinical stage	
I+II	86
III+IV	46

Supplementary Table S2: Association between BRF1 expression and clinicopathologic characteristics of patients with HCC (*n* = 133)

Clinical features	Number	BRF1 (-)	BRF1 (+)	*P*-value
Gender				0.279
Female	21	3	18	
Male	112	32	80	
Age (yrs)				0.63
< 50	62	15	47	
> 50	71	20	51	
HbsAg				0.187
Negative	22	3	19	
Positive	111	32	79	
Serum AFP (ng/ml)				0.028
< 400	76	26	50	
> 400	56	9	47	
Serum ALT				0.559
< 40	70	17	53	
> 40	62	18	44	
Tumor size (cm)				1.00
< 5	47	12	35	
> 5	86	23	62	
Recurrence				0.554
Absent	68	20	48	
Present	64	15	49	
TNM stage (AJCC)				0.218
Early (I-II)	86	26	60	
Advanced(III-IV)	46	9	37	

Supplementary Table S3: siRNA targets

Targets	Sequences
Mismatch	sense 5′UUC UCC GAA CGU GUC ACG Utt 3′<br>antisense 5′ACG UGA CAC GUU CGG AGA Att 3′
Mouse Brf1 siRNA (1)	**A**: sense 5′ ACC UUG AGA UUG ACA GAU A dTdT 3′:<br>antisense 5′ UAU CUG UCA AUC UCA AGG UdTdT 3′;<br>**B**: sense 5′ AAG CAC UGC CCC ACU UAU UUG dTdT 3′<br>antisense 5′ CAA AUA AGU GGG GCA GUG CUU dTdT 3′<br>**C**: sense: 5′ AAG CAC UGC CCC ACU UAU UUG dTdT 3′<br>antisense: 5′ CAA AUA AGU GGG GCA GUG CUU dTdT3′
Human Brf1 siRNA (2)	**A**: sense 5′ GGA AGA UCU GUU GUU ACU U 3′<br>antisense 5′ AAG UAA CAA CAG AUC UUC C 3′<br>**B**: sense 5′ CCC GUG CCU GUA UAU UCC A 3′<br>antisense 5′ UGG AAU AUA CAG GCA CGG G 3′<br>**C**: sense 5′ GAG CAU AGC GCC AGU GCC A 3′<br>antisense 5′ UGG CAC UGG CGC UAU GCU C 3′

Supplementary Table S4: Primer Sets for Quantitative RT-PCR

Target	Primers	Annealing Temperature
Pre-tRNA^{Leu} (3)	(F) 5′-GTC AGG ATG GCC GAG TGG TCT AAG-3′<br>(R) 5′-CCA CGC CTC CAT ACG GAG AAC CAG AAG ACC C-3′	61°
5S rRNA (4)	(F) 5′ GGC CAT ACC ACC CTG AAC GC 3′<br>(R) 5′ CAG CAC CCG GTA TTC CCA GG 3′	61°
Mouse Brf1(4)<br>Human Brf1 (4)	(F) 5′ GGA GCA GAG CCA ATC AAG CCA -3′<br>(R) 5′ CAT CAC CAT CAC AGC CGT AAT C -3′<br>(F) 5′ CCT CGG GCC TCT GCG GAG CAG -3′<br>(R) 5′ TCA TCA ATG GTC AAC TGA CTG GTG G -3′	62°<br>60°
GAPDH (4)	(F) 5′-TCC ACC ACC CTG TTG CTG TA-3′<br>(R) 5′-ACC ACA GTC CAT GCC ATC AC-3′	61°

Abbreviations: (F) = forward, (R) = reverse.

SUPPLEMENTARY REFERENCES

1. Johnson SA and Johnson DL. Enhanced RNA polymerase III-dependent transcription is required for oncogenic transformation. *J Biol Chem*. 2008; 283:19184–19191.
2. Zhong Q, Shi G, Zhang Q, Zhang Y, Levy D, Zhong S. Role of phosphorylated histone H3 serine 10 in DEN-induced deregulation of Pol III genes and cell proliferation and transformation. C*arcinogenesis*.2013; 34: 2460–2469.
3. Crighton, D., Woiwode, A., Zhang, C., Mandavia, N., Morton, J.P., Warnock, L.J. *et al*. p53 represses RNA polymerase III transcription by targeting TBP and inhibiting promoter occupancy by TFIIIB. *EMBO J*. 2003; 22: 2810–2820.
4. Zhong S. and Johnson DL. The JNKs differentially regulate RNA polymerase III transcription by coordinately modulating the expression of all TFIIIB subunits. *Proc Natl Acad Sci U S A*. 2009; 106:12682–12687.
